# Advances in the Application of Cold Plasma Technology in Foods

**DOI:** 10.3390/foods12071388

**Published:** 2023-03-24

**Authors:** Elisa Sainz-García, Fernando Alba-Elías

**Affiliations:** Department of Mechanical Engineering, University of La Rioja, C/San José de Calasanz 31, 26004 Logroño, Spain

In the last two decades, non-thermal processing technologies have gained widespread attention from the food industry, which is interested in mild and effective processes. These alternative technologies may increase functionality and shelf-life, reducing the negative impact on food nutrients and natural flavor. Some of the most successful non-thermal methods are high-pressure processing, ultrasound, pulsed electric field, ultraviolet light, high-intensity pulsed light, gamma irradiation, and, most recently, cold plasma (CP).

In the last decade, this novel approach has shown promising results as a disinfectant of food products and food contact surfaces. The novelty of this technology lies in its versatility of production and application (direct or indirect plasma, plasma treated water, functional coatings, etc.) and its non-thermal nature, since the quality attributes of food products have not been negatively affected.

This Special Issue is composed of five different works—one review paper, and four research articles—written by a group of international researchers to provide up-to-date research on different dimensions of the innovative advances in CP research achieved by the food industry.

Biofilms are an important problem in food industries because they enable bacteria to adhere to a wide variety of materials commonly used in food-contact surfaces (plastics, stainless steel, etc.) and they provide bacteria with protection against antimicrobial agents and stressful environments. Muro-Fraguas et al. [1] from Spain studied the durability of a plasma-polymerized coating with anti-biofilm activity over stainless steel. The results confirmed the effectiveness of the coating for the inhibition of multi-strain Listeria monocytogenes biofilm formation after five sanitization cycles with peracetic acid and sodium hypochlorite solutions.

Related to food safety, CP applications could be designed to suppress the negative effects that potentially affect consumer health and preferences such as the stability of the quality-determining ingredients or the permanency of non-desirable residues in CP treated foods. Tarabová et al. [2], developed an interesting work regarding the CP processing of fresh apple juice. This treatment showed no change in the physico-chemical parameters of the juice (pH, conductivity, color, transmittance and Brix degree), a safe nitrate concentration and virtually no effect on the individual natural components of the juice (sugar, organic acids and polyphenols). The CP treatment extended the juice shelf-life by up to 26 days if refrigerated, which represents a promising application potential in food technology.

Another field of interest in the food industry is the curing process in meat products for the development of color and flavor, suppression of microbial growth, and inhibition of oxidation in meat products. However, increasing concerns about the use of synthetic additives have led to an increase in consumer demands to use natural nitrite sources. Accordingly, Chen et al. [3] used CP technology as an alternative in the curing of roasted lamb. This treatment was effective and enhanced the sensory attributes of the roasted lamb.

Due to the rapid changes in consumer lifestyle, there has been an exponential increase in the demand for Ready-To-Eat (RTE) food products. Failing to maintain cold storage conditions during storage and/or transportation and the manipulation of RTE food in kitchens should be highlighted as potential risks, as they lead to the possible proliferation of bacteria or cross-contamination with other pathogen sources. However, most of these foods are consumed without any culinary preparation to eliminate microbial loads, which could be a source of foodborne outbreaks, as other authors have reported. To deal with this problem, Calvo et al. [4] evaluated the effectiveness of CP for the inactivation of different bacteria inoculated on the surface of different slices of RTE foods (“chorizo”, salami, bacon, smoked salmon, tofu and apple). They concluded CP is a successful non-thermal technique for the disinfection of RTE products, with different antimicrobial mechanisms for Gram-positive and Gram-negative bacteria.

Finally, a review article was written by Scholtz et al. [5]. The authors’ review aimed to present the current state-of-the-art of CP for the microbial decontamination of different cereals. CP seems to be an effective treatment to fight bacteria and fungi without any significant decrease in cereal quality.

In summary, the Special Issue “Advances in the Application of Cold Plasma Technology in Foods” demonstrates that CP is a promising technology, with different applications in the food industry.

I am very grateful to the authors for generously sharing their scientific knowledge and expertise with others through their contributions to this Special Issue.
